# Hepatocyte Mitochondrial Dynamics and Bioenergetics in Obesity-Related Non-Alcoholic Fatty Liver Disease

**DOI:** 10.1007/s13679-022-00473-1

**Published:** 2022-05-02

**Authors:** Aigli-Ioanna Legaki, Ioannis I. Moustakas, Michalina Sikorska, Grigorios Papadopoulos, Rallia-Iliana Velliou, Antonios Chatzigeorgiou

**Affiliations:** 1 Department of Physiology, Medical School, National and Kapodistrian University of Athens, 75 Mikras Asias Str, 11527 Athens, Greece; 2 Institute for Clinical Chemistry and Laboratory Medicine, University Clinic Carl Gustav Carus, Technische Universität Dresden, Fetscherstrasse 74, 01307 Dresden, Germany

**Keywords:** Liver, NAFLD, NASH, Mitochondrial dysfunction, Mitochondrial bioenergetics, Energy metabolism

## Abstract

***Purpose of the Review*:**

Mitochondrial dysfunction has long been proposed to play a crucial role in the pathogenesis of a considerable number of disorders, such as neurodegeneration, cancer, cardiovascular, and metabolic disorders, including obesity-related insulin resistance and non-alcoholic fatty liver disease (NAFLD). Mitochondria are highly dynamic organelles that undergo functional and structural adaptations to meet the metabolic requirements of the cell. Alterations in nutrient availability or cellular energy needs can modify their formation through biogenesis and the opposite processes of fission and fusion, the fragmentation, and connection of mitochondrial network areas respectively. Herein, we review and discuss the current literature on the significance of mitochondrial adaptations in obesity and metabolic dysregulation, emphasizing on the role of hepatocyte mitochondrial flexibility in obesity and NAFLD.

***Recent Findings*:**

Accumulating evidence suggests the involvement of mitochondrial morphology and bioenergetics dysregulations to the emergence of NAFLD and its progress to non-alcoholic steatohepatitis (NASH).

***Summary*:**

Most relevant data suggests that changes in liver mitochondrial dynamics and bioenergetics hold a key role in the pathogenesis of NAFLD. During obesity and NAFLD, oxidative stress occurs due to the excessive production of ROS, leading to mitochondrial dysfunction. As a result, mitochondria become incompetent and uncoupled from respiratory chain activities, further promoting hepatic fat accumulation, while leading to liver inflammation, insulin resistance, and disease’s deterioration. Elucidation of the mechanisms leading to dysfunctional mitochondrial activity of the hepatocytes during NAFLD is of predominant importance for the development of novel therapeutic approaches towards the treatment of this metabolic disorder.

## Introduction

The liver has been characterized as the organism’s metabolic center, since it is responsible for regulating a number of biological processes, including the maintenance of energy homeostasis, production of several biomolecules such as bile and vitamins, and scavenging of harmful endogenous and exogenous metabolites [1, 2]. The orchestration of such procedures demands excessive energy requirements; therefore, the presence of operative mitochondria in hepatocytes is indispensable [1]. Indeed, hepatocytes, constitute 70–85% of the total liver mass and among cell populations residing in the liver, they are the most prone to cellular impairment. Disruption of their capacity to operate properly can lead to numerous pathological conditions, including obesity-related insulin resistance and non-alcoholic fatty liver disease (NAFLD) [1–4]. To abstain such afflictions, hepatocytes require a sufficient amount of adenosine triphosphate (ATP), necessary for the proper execution of biological processes. Hence, mitochondria are found in great numbers in hepatocytes and consist a crucial part in the metabolism of hepatocytes, as they constitute the principal site for oxidative phosphorylation (OXPHOS) and fatty acid oxidation (FAO), leading to ATP synthesis [2]. Due to their highly dynamic nature, mitochondria can undergo functional and structural adaptations to meet the metabolic requirements of the cell through biogenesis, namely the growth and split of preexisting mitochondria, as well as through the opposite processes of fission and fusion, viz the separation and merge of mitochondria respectively [5]. Disruption in mitochondrial function tends to provoke and aggravate obesity-related metabolic dysregulation and likely contributes to the advancement of NAFLD [6]. Besides, NAFLD is thought as the metabolic manifestation of obesity-related metabolic dysregulation in the liver [7].

Evidence suggests that the emergence of obesity can be attributed to dysregulation of mechanisms of energy homeostasis, rather than simply evolving from excess caloric intake, implying that both obesity and NAFLD could be considered as “mitochondrial diseases” [8, 9]. NAFLD is strongly associated with obesity, with the latter acting as an autonomous risk factor for developing the first. A 3.5-fold higher risk for NAFLD advancement has been proposed for obese individuals compared to non-obese, while body mass index (BMI) appears to be responsible for NAFLD susceptibility in a “dose-dependent” manner [10]. The pathophysiology of NAFLD is guided by the implications on lipid metabolism and other metabolic pathways present in obesity, which result in the accumulation of intrahepatic fat (liver steatosis). Epidemiologic data presented in numerous studies confirm the correlation between obesity and NAFLD. Accordingly, NAFLD prevalence in obese individuals is greater than that of the general population (25–30%), although it varies in different studies with respect to age and other predisposing factors, including type 2 diabetes [11].

In this review, we discuss the recent literature on the impact of mitochondrial adaptations in metabolic disorders, emphasizing on the role of hepatocyte mitochondrial flexibility in obesity and the initiation and advancement of NAFLD. To that end, the current review specifically focuses on liver mitochondrial adaptations across the spectrum of obesity initiated NAFLD, covering the fundamental as well as the latest research on this topic.

## Mitochondrial Adaptations: Mitochondrial Dynamics and Bioenergetics

Mitochondria are involved in various substrate catabolism processes, namely FAO (or β-oxidation) and tricarboxylic acid cycle (TCA) [2, 12]. Their primary physiological function is to generate energy in the form of ATP by performing OXPHOS, a process carried out in the electron transport chain (ETC) complexes. Specifically, important cellular metabolites such as pyruvate, amino-acids, and fatty-acids are carried forward into the TCA cycle for production of the reducing equivalents nicotinamide-adenine dinucleotide (NADH) and flavine-adenine dinucleotide (FADH2), which sequentially transfer their electrons to oxygen at the ETC [13]. Importantly, during this process, approximately 0.25–11% of the oxygen consumed by the mitochondrion is converted into superoxide and hydrogen, also known as reactive oxygen species (ROS), contributing to mitochondrial injury in several metabolic pathologies [14].

**Fig. 1:**
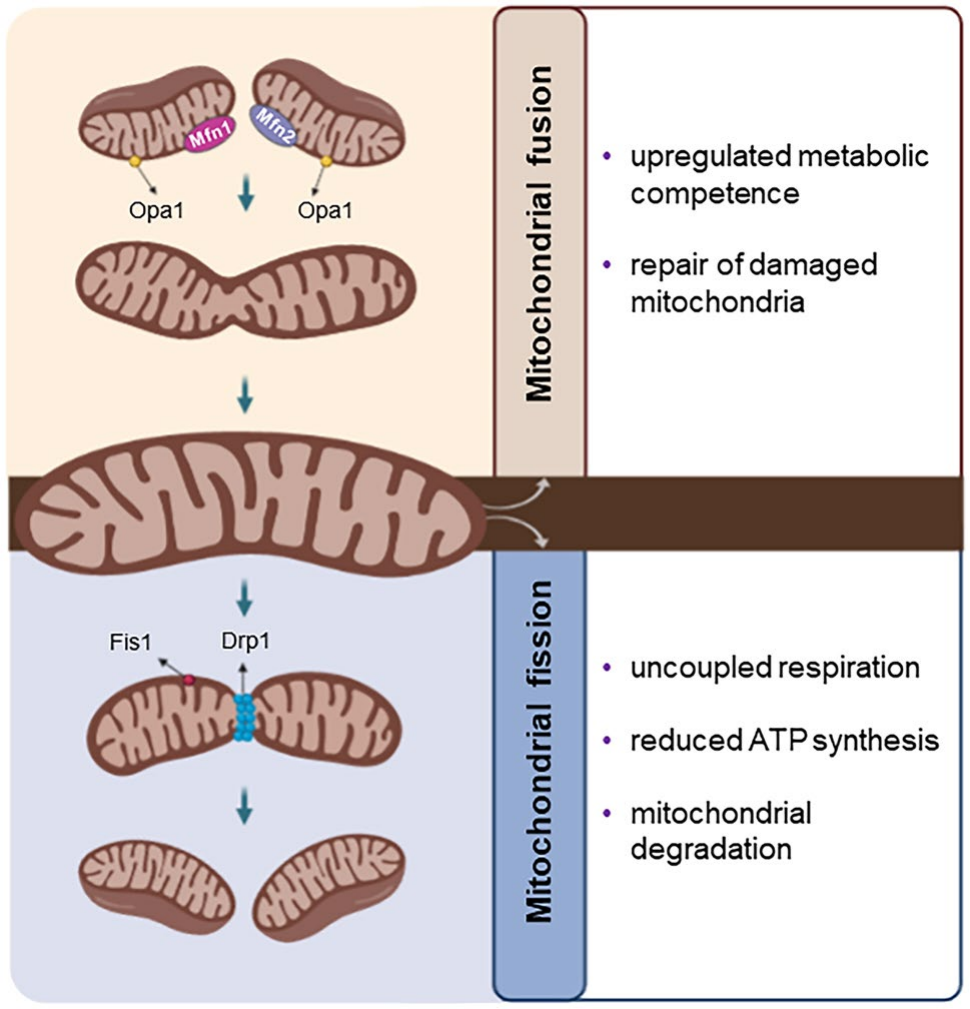
Mitochondrial dynamics: the process of fission and fusion. Mitochondrial fission is regulated by Drp1 and FIS1, which compress and separate mitochondrial tubules. Under conditions of low energy demand, fission facilitates uncoupled respiration, resulting to reduced ATP synthesis. Oppositely, during fusion, Mfn1/2 and OPA1 integrate the OMMs and IMMs. Mitochondrial fusion is stimulated by energy demand and stress and leads to upregulation of metabolic competence. Drp1, dynamin-like/related protein 1; FIS1, mitochondrial fission 1 protein; Mfn1, mitofusin 1; Mfn2, mitofusin 2; OPA1, optic atrophy 1; OMM; outer mitochondrial membrane; IMM, inner mitochondrial membrane

Mitochondria are assembled in a network of interconnected organelles, able to undergo morphological and functional adaptations to meet the metabolic requirements of the cell, following environmental signals [15]. These morphological changes can arise either as mitochondrial fission (fragmentation) or as fusion (connection). The equilibrium among these two opposite processes regulates mitochondrial number, size and positioning of mitochondria in the cytoplasm, is known as “mitochondrial dynamics” and is regulated by the membrane-remodeling proteins. During fission, dynamin-like/related protein 1 (Drp1) and mitochondrial fission 1 protein (FIS1) compress and separate mitochondrial tubules [16–19]. Oppositely, during fusion, mitofusins 1 and 2 (Mfn1/2) and optic atrophy-1(OPA1) integrate the outer (OMM) and inner (IMM) membrane of mitochondria, respectively [16, 18, 19]. Mitochondrial fusion is triggered from energy requirements and stress leading to an upregulation of metabolic competence and the repair of damaged mitochondrial fragments. In contrast, fission, triggered under relaxed conditions, facilitates uncoupled respiration, thus being related to reduce ATP synthesis. Fission is essential for mitochondrial degradation, where damaged parts of the mitochondria are in turn removed through mitochondrial autophagy, namely mitophagy [17, 20, 21] (Fig. 1). Importantly, mitophagy is of substantial significance in sustaining mitochondrial and cellular homeostasis under stress conditions, while dysregulated mitophagy is thought to have a pathological impact on the development of NAFLD [22–24].

### Mitochondrial Plasticity Assessment Methods

Mitochondrial plasticity, another term for mitochondrial adaptation, is directly associated with metabolic flexibility and can be characterized as an adaptive alteration in the mitochondrial content and function, depending on bioenergetic environmental conditions. The fundamental methodologies applied to study mitochondrial plasticity allow for the assessment of the ATP content under resting conditions or ATP production levels caused by metabolic alterations in whole tissues or cells. In principle, such methods involve measuring phosphorus metabolites primarily ATP or phosphocreatine via phosphorus (31P) magnetic resonance spectroscopy for measuring 31P-MRS in resting conditions or following induced metabolic alterations [25]. The OXPHOS rate constant is calculated by quantifying cellular oxygen depletion levels or isolated mitochondria under specific conditions of oxygen and existing energy substrate [26, 27]. In the liver, the submaximal ADP-stimulated OXPHOS activity can be quantified through 31P-MRS following intravenous or oral fructose intake, which triggers an intracellular ATP reduction via the activity of fructokinase [28–30]. Additionally, key observations can be attained via in vitro quantification of the activity of the ETC enzymes, ROS, and lipid peroxidation derivatives as a result of mitochondrial respiration, and antioxidant enzymes including catalase activity, decreased/oxidized glutathione ratio [12, 31, 32].

Methods applied for measuring the basal and maximum OXPHOS activity following ADP stimulation essentially require for high-resolution respirometry. The oxidative capability can be measured under specific energy conditions involving noncanonical, saturating energy substrate flows in the β-oxidation and Krebs cycle [33, 34]. This method allows for fundamental physiological properties of the mitochondrial respiratory chain to be calculated without the contradictory outcomes of differential oxygen or energy substrate transfer which could affect specific in vivo procedures. However, it relies its efficiency on the number of available biopsies which allow to accumulate substantial amounts of tissue to enable systematic mitochondrial analyses.

The evaluation of mitochondrial plasticity in the context of bioenergetics, as well as in the context of dynamics, can be achieved using quantitative PCR (qPCR) to quantify the expression levels of genes implicated to either mitochondrial bioenergetic and metabolic signaling or fission and fusion [35–41]. The morphology of mitochondria is not only fundamental for the maintenance of their optimal functionality but also critically associated to the metabolism of energy and OXPHOS function. Mitochondria morphology and dynamics can be assessed through the examination of tissue sections via electron microscopy (EM). Such methods allow for the visualization of ultrastructural alterations in mitochondrial characteristics, including loss of inner membrane and cristae can be observed, along with changes in mitochondria size (larger or shorter mitochondria) or granules, peroxisome proliferation, and mitochondrial fission or fusion [37, 39, 42–45]. Mitochondrial fission and fusion can also be evaluated using Immunofluorescence and Immunoblot analysis of fusogenic mediators including Mfn1, Mfn2, and OPA1 and fission operators such as Drp1 [46].

## Mitochondrial Adaptations in Health and Disease

### General Aspects

Mitochondrial adaptability to cellular needs and stress conditions is achieved via activation of advanced intracellular mechanisms. The mitochondrial nuclear crosstalk is of predominant importance in this response and involves the generation of various mitochondrial stress signals and nuclear stress response pathways. Mitochondria release metabolites such as acetyl-CoA and “mito-stress” signals, namely ROS, with a form of retrograde signaling, namely a mitochondria-to-nucleus signaling process that causes alterations in nuclear gene expression. The nucleus responds to these signals by activating stress-induced transcriptional programs, such as the AMP-activated protein kinase (AMPK) signaling. This in turn activates peroxisome proliferator-activated receptor γ coactivator 1-α (PGC-1α) and other transcriptional regulators, including cAMP-response element binding protein (CREB), nuclear factor-κB (NF-κB), and p53. These specific nuclear transcriptional responses are responsible for the repair or removal of damaged mitochondrial DNA (mtDNA) by fusion or fission and a switch towards glycolytic/lactate metabolism. In that way, ATP production is adjusted to meet the energy requirements of the cell [47–49]. For instance, when ATP levels decrease, the aforementioned “mitochondrial quality control” reinforces the cellular bioenergetic competence by enhancing the mitochondrial network via mitochondrial biogenesis and fusion, thus provoking the production of more ATPs [47, 50, 51].

Any dysfunction in this mitochondrial response mechanism is possible to affect cellular function rendering the cell more vulnerable to exogenous stressors, including oxidative stress as well as hypoxic conditions [47, 50, 52]. Gene mutations necessary for mitochondrial fusion, such as OPA1 or Mfn2, can affect multiple tissues, leading to diseases such as autosomal dominant optic atrophy (DOA) and Charot-Marie-Tooth type 2A (CMT2A) respectively [53–55]. Besides, abnormalities in mitochondrial structure and functionality affect the emergence and progression of neuromuscular and neurodegenerative disorders, in particular Parkinson’s, Alzheimer’s, and Huntington’s diseases [56]. Additionally, mitochondrial oxidative dysfunctions occur as secondary effect in the course of several diseases, including cardiovascular, gastrointestinal, skin disorders, and cancer [47, 57–59]. Importantly, mitochondria are associated with cancer advancement, since they enable the capacity of cancer cells to respond to the rapidly changing environmental conditions [47, 52, 59]. Finally, several germline and somatic mtDNA mutations are thought to play a key role in tumor enlargement in hemopoietic, prostate, breast, and renal cancer [60].

### Mitochondrial Adaptations in Obesity and Metabolic Dysregulation

During obesity, the excess nutrient input as well as the increased basal lipolysis taking place in adipocytes supplies hepatocytes with increased amounts of free-fatty acids (FFAs), subsequently leading to increased but still insufficient FAO and OXPHOS [61, 62]. This process of oversaturated FAO and OXPHOS inevitably induces mitochondrial dysfunction with an increase of ROS formation and endoplasmic reticulum (ER) stress, provoking mtDNA damage, lipid peroxidation, and likely cell death. Additionally, the stressed hepatocytes secrete inflammatory cytokines and chemokines as well as lipid peroxidation derivatives including malondialdehyde (MDA), resulting to the recruitment of immune cells and triggering hepatic inflammation and insulin resistance [58, 63, 64].

Several studies have reported abnormal mitochondrial activity during obese and/or insulin-resistant conditions. Reduced biogenesis and insufficient oxidative mitochondrial capacity are previously reported in adipose tissue of rodents and individuals with obesity. Houstis et al. have demonstrated that ROS production triggers insulin resistance in cultured 3T3-L1 adipocytes treated with dexamethasone or recombinant mouse TNF and in leptin-deficient ob/ob mice [65, 66]. Analysis of isolated mitochondria from rodents that followed a high-fat diet (HFD) was presented with diminished respiratory capacity along with elevated oxidative stress [67–69]. In addition, obese diabetic db/db and ob/ob mice displayed weakened respiratory capacity of mitochondrial complexes in liver homogenates [70, 71]. Data from obese and diabetic individuals indicate that skeletal muscle mitochondria exhibit lower muscle energy production capacities, decreased FAO and impaired mitochondrial OXPHOS [72, 73]. Moreover, smaller and shorter mitochondria along with elevated mitochondrial fission were observed in the skeletal muscle of obese mice, confirming the correlation between altered mitochondrial fission and insulin resistance in the skeletal muscle [74]. Mitochondrial impairment has also been implicated with elevated fission processes associated with ROS formation in the liver of db/db mice, in rat liver cell lines and H9c2 rat myoblasts treated with high glucose [70, 75]. On the contrary, other studies displayed unaltered or augmented hepatic oxidative activity of mitochondria in adult ob/ob mice as well as in insulin-resistant diabetic Goto-Kakizaki (GK) rats [76, 77]. The justification for these opposing results remains unidentified but might be associated with the variety of diets used, the range of obesity, and different experimental approaches for the evaluation of mitochondrial function.

## Hepatocyte Mitochondrial Adaptations in Obesity-Related NAFLD/NASH

### Hepatocytes, Obesity, and NAFLD Pathogenesis

The liver, as a regulator of lipid homeostasis, orchestrates the biosynthesis of new fatty acids and sterols, along with their subsequent allocation to other tissues, and their utilization as substrates for energy production [78]. When the balance between lipid gain and disposition is disrupted, fat accumulates in the liver, leading to hepatocyte metabolic dysregulation and liver steatosis. Hepatic fat accumulation is controlled by four important processes, namely the uptake of circulating lipids, FAO, de novo lipogenesis (DNL), and the release of lipids into the circulation in the form of very low-density lipoproteins (VLDL). When one or more of these pathways are dysregulated, hepatic lipid aggregation is induced, contributing to obesity and NAFLD pathogenesis [79].

The transport of circulating fatty acids into the hepatocytes is mainly facilitated by fatty acid transport proteins (FATP), cluster of differentiation 36 (CD36), as well as plasma membrane caveolins. Studies in mice have shown that knockdown of FATP2 or FATP5, two major hepatic FATP isoforms, along with selected deletion of fatty acid translocase protein CD36 in mouse genetic and HFD-induced steatosis models, results in hepatic lipid uptake reduction and amelioration of hepatic steatosis [80–82]. Similarly, when caveolin-1 (CAV1), a structural caveolae protein in the plasma membrane related to hepatic lipid translocation, is ablated in HFD mice, liver steatosis is reduced while hepatic gluconeogenesis is elevated [83]. The second pathway, namely FAO, takes place in mitochondria. It is controlled by peroxisome proliferator-activated receptor α (PPARα) and following its activation, FAO-related genes are transcripted [84]. In ob/ob mice, knockout of PPARα augmented obesity and hepatic steatosis due to decreased FAO [85]. Regarding hepatic lipid aggregation, Zhang et al. demonstrated that the lack of long chain acylCoA dehydrogenase (LCAD), an essential enzyme for mitochondrial FAO, predisposes mice to liver steatosis and insulin resistance [86]. Furthermore, in response to increased food intake, hepatic DNL is induced, which in turn is activated by upregulated insulin signaling and augmented glucose concentrations [87]. Finally, as TGs export from hepatocytes, mainly in the form of VLDL, hepatic lipid load decreases. During obesity and NAFLD, an increased export of VLDL occurs at the beginning of the disease. The latter is plateaued as the disease progresses, leading to escalated fat accumulation further perpetuating hepatic steatosis [88, 89].

### Hepatocyte Mitochondrial Bioenergetics in Obesity and NAFLD

**Fig. 2:**
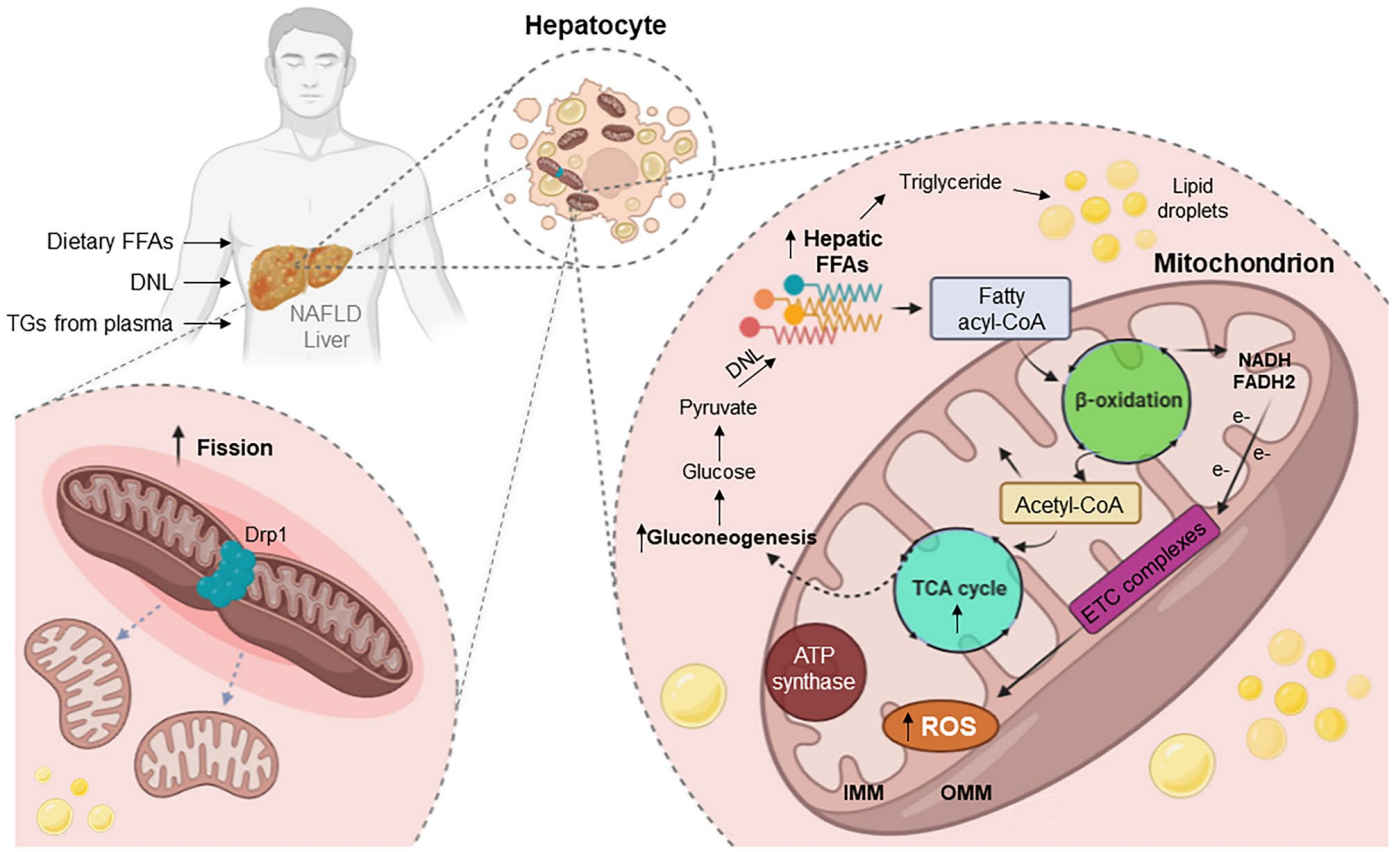
Hepatic mitochondrial adaptations in non-alcoholic fatty liver disease (NAFLD). In NAFLD, rapid accumulation of triglycerides (TGs) in the liver, due to high availability of free fatty acids (FFAs), and/or de novo lipogenesis (DNL) is associated with an elevated mitochondrial oxidative activity. The cytoplasmic FFAs are first converted into fatty acyl-CoA which is relocated to mitochondria to be decomposed via β-oxidation and produce acetyl-CoA. Increased FFA influx leads to insufficient hepatic β-oxidation and therefore, lipotoxic intermediates accumulate, triggering inflammation and disrupting insulin signaling. In contrast, the utilization of acetyl-CoA by the mitochondrial tricarboxylic acid (TCA) cycle continues unabated to meet the energetic demands of gluconeogenesis. Mitochondrial β-oxidation generates NADH and flavine-adenine dinucleotide (FADH2), the electrons (e-) of which are transferred to the electron transport chain (ETC). Disruption of the electron flow within the ETC induces leakage of electrons and the generation of reactive oxygen species (ROS), contributing to NAFLD progression, mainly by triggering hepatocyte stress and damage. Furthermore, when hepatocytes are exposed to excessive nutrient overload and FFAs, mitochondria become disintegrated through increased fission. DNL, de novo lipogenesis; TGs, triglycerides; OMM; outer mitochondrial membrane; IMM, inner mitochondrial membrane; ATP, adenosine triphosphate; Drp1, dynamin-like/related protein 1. Pathways and procedures that are increased are designated by **↑**

Hepatic energy homeostasis is mainly mediated by mitochondrial metabolic processes, encompassing β-oxidation, TCA cycle, ketogenesis, ETC activity, and ATP formation [90, 91]. During NAFLD, FFAs and TGs rapidly accumulate in the liver via the circulation and/or DNL and are associated with enhanced FAO and ROS formation, further contributing to NAFLD progression [2, 92, 93]. Specifically, under physiological conditions, the FFAs present in the cytoplasm are initially transformed into fatty acyl-CoA, which either remains in the cytoplasm to be used for esterification into TGs and can be secreted as VLDLs into the circulation or relocated to the mitochondria. Therein, disintegration follows through β-oxidation to compose acetyl-CoA [38, 94]. In obesity, increased FFA influx due to excess caloric intake leads to TGs aggregation in the form of lipid droplets in hepatocytes, a feature of NAFLD pathology [38, 90]. Besides, heightened FFA availability upregulates β-oxidation and consecutive production of NADH and FADH2 that serve as co-enzymes in the mitochondrial ETC, acting as electron transporters [38]. Hence, the electron flow within the ETC is overloaded and subsequently disrupted due to upregulation of proton leak, an over-reduction of the respiratory chain along with ROS generation (Fig. 2). This overproduction of ROS triggers the secretion of proinflammatory cytokines such as IL-1β and TNF by the hepatocytes, starting an endless loop of immune cell recruitment and inflammation, perpetuation of steatosis, and activation of hepatic stellate cells into profibrotic cells [6, 93, 95]. Besides, genomic- and mitochondrial-DNA damage can occur during this process, provoking hepatocyte senescence, which has been shown to aggravate NAFLD and progression to non-alcoholic steatohepatitis (NASH) [96, 97].

Several mitochondrial genomic and transcriptomic alterations have been reported during the whole phenotypic spectrum of NAFLD in both humans and rodents affecting the metabolic machinery of the mitochondria. Sookoian et al. have performed mitochondrial genome sequencing in the liver of NAFLD patients across the entire spectrum of the disease. Their findings revealed that the hepatic mtDNA of NAFLD patients harbors intricate mitochondrial genomes with a notably elevated mutation rate as compared with controls. Moreover, analysis of the entire hepatic mitochondrial genomes of patients with progressed fibrosis indicated that the severity of the disease is associated with increased number of hepatic mtDNA mutation-carrying variants encoding for proteins implicated in OXPHOS [98]. Other studies demonstrate downregulated expression of genes encoding for members of the OXPHOS system, resulting from epigenetic alterations taking place at the hepatic mtDNA [36, 37]. For instance, Pirola et al. provided evidence that increased hepatic mtDNA methylation affecting the transcriptional activity of mitochondrially encoded NADH dehydrogenase 6 (MT-ND6) participates in modulation of the histological severity of NAFLD leading to NASH [37]. Regarding the unrelated to obesity phenotype of NAFLD, a clear correlation of this lean phenotype and mitochondrial adaptations is missing [99]. However, Komatsu et al. described that hepatic steatosis occurs in patients afflicted with citrullinemia type 2 (CTLN2), an autosomal recessive disorder caused by a mutation in the gene encoding mitochondrial aspartate glutamate carrier 2 (SLC25A13) and these patients were not categorized as obese [100]. Furthermore, a study by Hakim et al. associates mitochondrial mutations with idiopathic NAFLD, as they report that non-obese NAFLD individuals harbor mutations in NADH dehydrogenase (ubiquinone) 1 beta subcomplex 3, (NDUFB3) gene, which encodes a subunit of respiratory chain complex I [101]. When NAFLD progresses to NASH, the metabolic flexibility of hepatic mitochondria including oxidative capacity and redox defenses ultimately fades [102, 103]. Koliaki et al. reported upregulation of OXPHOS effectiveness in hepatic mitochondria obtained from patients at early stages of NAFLD and significant downregulation in NASH patients [104]. Furthermore, it is demonstrated that NASH is correlated to genetic changes of hepatic cellular respirasome, involving high cytochrome b variations along with mtDNA damage, leading to severe respirasome supercomplex inadequacy and consequently resulting to cell death in high energy-demanding tissues [105]. Besides, other studies regarding obesity reported reduced hepatic ATP concentration in ob/ob mice and those of obese and diabetic individuals [106–108]. Similarly, Nair et al. demonstrated an inverted correlation between ATP hepatic stores and BMI, although the ATP reduction and recovery rates did not differ among obese and lean individuals upon fructose injection [29]. Additional data suggest that mice and patients with NASH exhibit downregulated ketogenesis, diminished mitochondrial respiration, mitochondrial rupture, and leakage [109]. On the contrary, during this progressive disease stage, the mitochondrial TCA cycle is hyperactive in order to respond to the high energy requirements [102, 104, 110]. Recent evidence of Grossini et al. demonstrates that the exposure of primary human hepatocytes to plasma from NAFLD patients leads to reduced hepatocyte viability and mitochondrial membrane potential in these cells as compared to those treated with plasma from healthy individuals, along with increased ROS and H_2_O_2_ production and triglyceride accumulation. Their results also presented that the plasma of NAFLD patients induced increased hepatocyte expression of peroxisome-proliferator-activating-ligand-receptor-γ (PPARγ), sterol-regulatory-element-binding-protein-1c (SREBP-1c), nuclear-factor-kappa-light-chain-enhancer of activated B cells (NF-kB), and NADPH oxidase 2 (NOX2), which are implicated to mitochondrial bioenergetic and metabolic signaling [35]. Ajaz et al. followed a mitochondrial functional and metabolomic approach in peripheral mononuclear blood cells (PBMCs) from patients with NAFLD at different fibrotic stages versus healthy individuals. They demonstrated that the progression of NAFLD is associated to mitochondrial abnormalities related to changes in metabolites of the urea cycle; specifically, reduced hepatic mitochondrial respiratory capacity and significant changes in five out of fourteen metabolites involved in urea cycle were observed in patients presenting with progressed fibrosis in comparison to mild/moderate fibrosis [111].

Although the findings above implicate mitochondrial dysfunction in NAFLD progression, opposing evidence exists in the literature. Studies examining the liver, muscle, and adipose tissue of mice with muscle- and liver-specific ablation of mitochondrial flavoprotein apoptosis inducing factor (AIF), inducing mitochondrial OXPHOS defects, have shown that abnormalities in mitochondrial respiratory activity may induce an insulin-sensitive metabolic condition, which protects against the adipogenic and diabetogenic effects of HFD [112]. Such evidence agrees with the phenotype reported in mice that specifically lack the mitochondrial transcription factor-A (TFAM) in muscle and adipose tissue which exhibited upregulated insulin susceptibility in both tissues, regardless of the inadequate mitochondrial respiratory system therein [113, 114]. Therefore, these reports impose that mitochondrial impairment might not be a trigger of NAFLD, suggesting instead that mitochondrial impairment might be a derivative, outcome of the disease’s advancement [115].

Another important component of mitochondrial bioenergetic adaptation in NAFLD is the transfer of a portion of excess energy intermediates in the ineffective cycle of uncoupled respiration. In preclinical studies, mitochondrial uncoupled respiration appeared increased due to the upregulation of the uncoupling protein 2 (UCP-2) in hepatocytes obtained from an obese rodent model. The in vitro exposure of rat hepatocytes to lipid emulsions resulted in augmented UCP-2 expression, possibly through mechanisms mediated by excess ROS production [106, 116]. Morris et al. demonstrated that a short-term HFD treatment on rats predisposed to obesity can lead to decreased metabolic adaptability and liver mitochondrial respiratory capacity which is effectively averted by hepatic overexpression of peroxisomal proliferator-activated receptor-γ coactivator-1α (PGC-1α) [117]. In human NAFLD, this phenomenon culminates in the established NASH environment and is also mediated by upregulated UCP-2 expression, which is consistent with indications of severe oxidative stress and injury [104, 118]. This illustrates that heightened uncoupled respiration in progressed NAFLD may act in a protective manner against the energetic overstrain and subsequent redox imbalance of ETC complex. Nevertheless, this comes at the cost of decreased bioenergetic effectiveness, possibly accounting for the diminished energy-producing capacity of the liver in NASH, making hepatocytes more susceptible to severe energy-demand challenges, such as ischemic injury [118, 119].

### Hepatocyte Mitochondrial Dynamics in Obesity and NAFLD

Hepatocyte mitochondrial dynamics is a fundamental mechanism of the adjustment of a cell to its metabolic requirements. When nutrient availability changes occur, mitochondria are subjected to coordinated fission and fusion cycles to sustain energy homeostasis. In rich-nutrient environmental conditions, mitochondria fragment to prevent energy waste, while reducing bioenergetic efficiency and augmenting mitochondrial uncoupling, which results to a simultaneous increase of nutrient storage [38, 74, 120]. On the contrary, under nutrient deprivation conditions, hepatic mitochondria remain elongated through the process of increased fusion [18]. Of note, when hepatocytes are exposed to high levels of cellular stress along with excessive nutrient overload and FFAs, a common phenomenon in obesity and NAFLD, the mitochondrial network becomes more disintegrated through increased fission [18, 19] (Fig. 2). The segregated mitochondria are frequently depolarized, a phenomenon caused by diminished respiratory capacity, enabling mitophagy to occur, which is a highly selective autophagic clearance of defective and dysfunctional mitochondria [121].

Numerous in vivo and in vitro studies highlight changes pertinent to mitochondrial fusion and fission during obesity and NAFLD. Evidence from in vitro studies suggests that when hepatocytes are treated with palmitate, their mitochondria become fragmented, lose their transmembrane potential, while there is a cytochrome c release in the cytoplasm and elevated ROS activity [122, 123]. It has been also demonstrated that protein expression of Drp1 is increased in animal models of NAFLD, indicating mitochondrial fragmentation [38–40]. Analysis made with electron microscopy exhibited higher mitochondrial fission in the livers of animals subjected to HFD, as well as heightened hepatocyte lipolysis [39, 42]. Moreover, by using HFD-feeding in mice that express the dominant-negative fission mutant DLP1-K38A in a doxycycline-inducible manner, Galloway et al. showed that transgenic inhibition of mitochondrial fission was protective against liver steatosis, alleviating HFD-induced oxidative stress and hepatic damage as well. This suggests a mechanistic role of mitochondrial fission in controlling hepatic lipid regulation and oxidative stress implicated with NAFLD [39]. Takeichi et al. showed that hepatic deletion of mitochondrial fission factor (MFF) stimulates ER stress and diminishes the secretion of triaglycerol in the liver both in vivo and in vitro. They also presented that mice lacking MFF in hepatocytes (MffLiKO mice) are more prone to NASH phenotypes caused by HFD than control mice, mainly due to the hepatic activation of apoptosis from ER stress and the suppression of triglyceride excretion from hepatocytes. Thus, their research brings new information for the implication of mitochondrial fission in NASH development [124].

Oppositely, a decline in mitochondrial fusion has been reported during NAFLD in vitro and in vivo. The expression of Mfn1 is reduced in hepatocytes of HFD-fed mice and is associated with steatohepatitis [41]. Importantly, a pro-inflammatory factor, CXCR3 expressed by hepatocytes, stellate cells along with a plethora of immune cells of the liver microenvironment induces a reduction of the protein levels of Mfn1, thereby inducing mitochondrial deterioration in animal models of NASH [40]. Palmitate treatment in hepatocytes induces downregulation of Mfn2 in both transcript and protein levels [123]. When Mfn2 was ablated specifically in mouse livers, hepatic ER activity was increased, leading to metabolic abnormalities, along with deteriorated glucose tolerance and insulin resistance in mice subjected to HFD [125]*.* Moreover, diminished Mfn2 levels are detected in the liver of NASH individuals and in NAFLD/NASH murine models. Interestingly, hepatic Mfn2 deletion promoted inflammation, triglyceride concentration, fibrosis, and hepatic cancer in mouse models of NASH, while adenovirus-mediated re-expression of Mfn2 in liver-specific Mfn2 knockout mice led to disease alleviation [126, 127]. These data demonstrate the importance of Mfn2 in NAFLD pathophysiology.

Regarding the mitochondrial structural morphology, it has been reported that hepatic mitochondria from NAFLD patients have circuit geometry, due to cristae loss. As the disease progresses, patients develop megamitochondria with paracrystalline inclusion bodies, indicating either a protective or a degenerative response to injury [43, 44]. These morphological deformations are a consequence of mitochondrial dynamics’ dysregulation in NAFLD patients, mainly attributed to disturbed ATP homeostasis due to impaired function of the mitochondrial ETC [44]. Similar modifications were observed in animal models of diet-induced obesity, where hepatic mitochondria appeared to have a rounder and shorter shape, with lack of cristae, while indications of swelling and matrix compression were also present [45]. Of note, mitochondrial ultrastructural modifications were prevented in HFD mice following a systematic strength training program versus sedentary HFD mice [128]. Although there is evidence associating alterations in mitochondrial integrity and elevated oxidative stress with obesity and NAFLD pathogenesis, additional investigation is necessary for the elucidation of the precise implication of mitochondrial pathophysiology into disease initiation and advancement. Tables 1 and 2 present major animal and human studies on mitochondrial adaptations in NAFLD.

**Table 1:** Major human studies on mitochondrial adaptation in NAFLD

**First author, year, reference**	**Cohorts**	**Tissue samples** **Cell lines**	**Methods**	**Key results**	**Challenges/limitations**
**Sookoian, 2016 **[98]	-Controls (8) and NAFLD patients (20) -Patients with NASH at different stages (10) and patients with moderate of advanced fibrosis (14) -Unrelated patients with NAFLD (NAFL = 62, NASH = 76) and healthy individuals (100)	Liver biopsies Serum samples PBMCs	Deep coverage whole genome sequencing in hepatic mitochondrial genome with NGS	-Hepatic mtDNA of NAFLD patients harbors intricate mitochondrial genomes with a notably ↑ mutation rate versus controls -In patients with ↑ fibrosis the severity of NAFLD is associated with ↑number of OXPHO-related hepatic mtDNA mutation-carrying variants	-Using NGS, the number of reads could probably limit the sensitivity to detect low frequency heteroplasmic variants and somatic mutations -Mitochondrial genetics differ from nuclear genetic -No functional data to support the effect of missence mutations
**Pirola, 2013 **[37]	Controls (18) NAFLD patients (45)	Liver biopsies	-Biochemical evaluation -Histopathological evaluation -Bisulfite treatment of DNA and methylation specific PCR -qPCR	-↑ hepatic mtDNA methylation affecting the transcriptional activity of MT-ND6 participates in modulation of histological severity of NAFLD leading to NASH	None
**Koliaki, 2015 **[104]	Obese IR patients w/o NAFLD (18) Obese IR patients with NAFLD (16) Obese IR patients with NASH (17) Lean individuals (12)	Liver biopsies Serum samples	-Metabolic characterization -Histology -Immunoblotting -Mitochondrial respiration evaluation -Oxidative stress evaluation -qPCR	-↑ OXPHOS effectiveness in liver mitochondria obtained from early stages of NAFLD patients -↓ OXPHOS effectiveness in liver mitochondria of NASH patients	-Insulin infusion -FCCP-induced maximum uncoupled respiration -ADP-stimulated respiration -There is no standard method for associating mitochondrial function to mitochondrial content
**Pirola, 2021 **[105]	NAFLD patients (252)	Liver biopsies	-MT-CYB sequencing and analysis of mtDNA damage -Global liver transcriptome analysis	-NASH correlation with genetic changes of liver cellular respirasome (↑ cytochrome b variations, mtDNA damage) resulting to severe respirasome supercomplex inadequacy and cell death	Modest sample size
**Grossini, 2021 **[35]	Controls (12) NAFLD patients (12)	Liver biopsies Plasma samples Huh7.5 cell line	-Hepatocyte isolation from human liver biopsies -Co-culture of Huh7.5/primary human hepatocytes by using Transwell inserts -Mitochondrial ROS, ROS-Glo H202 quantification -Immunoblotting	-Exposure of primary human hepatocytes to plasma from NAFLD patients results to ↓ hepatocyte viability and ↓ mitochondrial membrane potential, ↑ ROS, ↑ H202 production compared to those treated with plasma from healthy individuals	None
**Ajaz, 2021 **[111]	Controls (10) NAFLD patients with fibrosis (10) NAFLD patients with severe fibrosis (10)	PBMCs	-Mitochondrial functional analysis with Seahorse XFp In PBMCs -Global metabolomics -ELISA	-NAFLD progression is associated with mitochondrial dysfunctions related to changes in metabolites of the urea cycle (↓ hepatic mitochondrial respiratory capacity, significant changes in 5 out of 14 metabolites of the urea cycle in patients with progressed fibrosis in comparison to mild/moderate fibrosis)	-Limited number of samples -Lack of liver tissue to measure gene expression of the urea cycle enzymes
**Nair, 2013 **[29]	Healthy individuals with varying BMI (19)	Liver biopsies	-MR Spectrometry	-↓ liver ATP stores are more common in overweight and obese individuals	Modest sample size

**Table 2:** Major Animal studies on mitochondrial adaptation in NAFLD

**First author, year, reference**	**Animal models**	**Tissue, samples** **Cell lines**	**Methods**	**Key results**	**Challenges/limitations**
**Gao, 2015 **[85]	-ob/ob mice -PPARa-deficient ob/ob mice	Liver	-Glucose tolerance and insulin resistance -Biochemical assays -Histology, immunohistochemistry -Immunoblotting -Northen blotting and qPCR	-Knockout of PPARa ↑ obesity and ↑ hepatic steatosis due to ↓ FAO	None
**Zhang, 2007 **[86]	LCAD-/- mice WT mice	Liver	-Hyperinsulinemic-Euglycemic clamp -Biochemical assays -qPCR	-Lack of LCAD predispose mice to liver steatosis and insulin resistance	None
**Pospisilik, 2007 **[112]	Liver- and Muscle-Specific AIF -/- mice	Liver muscle Adipose tissue	-Glucose and insulin tolerance tests -Hyperinsulinemic clamp -Indirect calorimetry -Immunoblotting -Mircoarray analyses -Determination of intracellular metabolites -Histology -Mitochondrial DNA quantification	-Mitochondrial dysfunctions in respiratory activity may induce insulin-sensitive metabolic condition, protecting against the adipogenic and diabetogenic effects of HFD in muscle- and liver-specific AIF knockout mice	None
**Galloway, 2014 **[39]	Transgenic mice expressing DLP1-K38A in a doxycycline-inducible manner	Liver	-Histology and immunohistochemistry -Mitochondrial morphology assessment -Isolation of Primary hepatocyte and treatments -Oxygen consumption analyses	-Transgenic inhibition of mitochondrial fission is protective against hepatic steatosis, ameliorating HFD-induced oxidative stress, and liver damage, suggesting a mechanistic role of mitochondrial fission in regulating hepatic lipid regulation and oxidative stress associated with NAFLD	None
**Takeichi, 2021 **[124]	Mice with hepatocyte-specific deletion of MFF (MffLi -/- mice)	Liver	-Isolation of primary hepatocytes isolated from MffLiKO -Metabolic and biochemical evaluations -qPCR -Immunoblotting -Mitochondria isolation experiments	-Hepatic deletion of mitochondrial fission factor (MFF) stimulates ER stress and ↓ the secretion of triglycerides in the liver -MffLiKO mice are more susceptable to NASH phenotypes caused by HFD than control mice	Further studies needed to delineate the correlation of mitochondrial fission with the regulation of glucose metabolism
**Du, 2017 **[40]	CXCR3-/- mice WT mice		-TEM -Mitochondria isolation from liver, Mitochondria membrane potential, mtDNA damage analysis -Immunoblotting, Immunofluorescence -FACS -qPCR -siRNA transfection for CXCR3 knockdown -ATP measurement	-CXCR3 ↓ the protein levels of Mfn1, thereby causing mitochondrial deterioration in animal models of NASH	None
**Sebastian, 2012 **[125]	Liver-specific Mfn2 KO mice	Liver Skeletal muscle Adipose tissue	-Isolation of mouse hepatocytes -High-resolution respirometry with the Oxygraph-2 k and Seahorse Bioscience XF	-Specific hepatic ablation of Mfn2 resulted to ↑ hepatic ER activity, leading to metabolic dysregulations, deteriorated glucose tolerance and insulin resistance in HFD-mice -Mfn2 coordinates mitochondria and ER function, leading to modulation of insulin signaling and glucose homeostasis in vivo	None
**Piacentini, 2018 **[42]	TG2-/- mice WT mice	Liver Serum samples	-Metabolic and biochemical evaluations -Histopathological analysis -Transmission electron ultrastructural analysis -Immunoblotting	-↑ mitochondrial fission when subjected to HFD -TG2 activation may offer protection in the context of NAFLD	None

## Conclusion and Future Perspectives

Emerging evidence illustrates that alterations in hepatic mitochondrial dynamics and bioenergetics hold a substantial role in the pathogenesis of NAFLD [2, 21, 104, 129]. When the fatty acid influx is elevated in hepatocytes, as it occurs during obesity and NAFLD, oxidative stress takes place as consequence of ROS overproduction, causing mitochondrial dysfunction. As a result, mitochondria become ineffective and disengaged from respiratory chain activity and ATP formation, further favoring hepatic fat accumulation, while leading to liver inflammation and insulin resistance in a ROS-dependent manner. These phenomena lead to subsequent hepatocyte dysfunction or damage and trigger the transition of NAFLD to NASH by parallel activation of stellate cells [39, 122, 123, 130, 131].

Therapeutic strategies diminishing the oxidative burden are able to reinforce the mitochondrial capacity for ATP generation and can suppress mitochondrial fission, thus attenuating or even reversing the advancement of NAFLD [1, 39]. Such approaches include anti-diabetic drugs such as thiazolidinediones, which are synthetic peroxisome proliferator-activated receptor gamma (PPAR-γ) ligands [132, 133]. For instance, pioglitazone, a widely known PPARγ agonist, inhibits cytochrome c leakage, normalizes the mitochondrial transmembrane potential, prevents ROS production, and induces the activation of the ETC complexes, and therefore could be used for NAFLD treatment as well [134]. Along this line, metformin, a first-line diabetic drug, has the capacity to stimulate the AMPK signaling pathway and enhance mitochondrial fission in mice following HFD, leading to upregulated mitochondrial respiration, stabilized mitochondrial membrane potential and increased ATP levels [135, 136]. Besides, angiotensin II type 1 receptor (ATIR) antagonists have been observed to have beneficial impact in rodent models of NAFLD, as these agents could augment mitochondrial biogenesis or protect mitochondria from oxidative stress [137]. Additionally, irisin, a recently identified hormone secreted by muscle cells, and resolvin D1 (RvD1), an anti-inflammatory and antioxidant lipid mediator, hinder mitochondrial fission and protect against liver ischemia–reperfusion injury [138, 139].

Therapeutic approaches directly targeting the mitochondria machinery have shown great potential against NAFLD. Specifically, therapeutic targeting of methylation-controlled J protein (MCJ) in the liver, an endogenous negative regulator of the respiratory chain complex I, efficiently ameliorated liver lipid accumulation and fibrosis in multiple NAFLD mouse models. Specifically, blockage of MCJ by utilizing nanoparticle- and GalNAc-formulated siRNA heightened hepatocyte capacity to mediate FAO and decreased lipid accumulation, resulting in diminished hepatocyte damage and fibrosis [140]. Moreover, pharmacological inhibition of Drp1 blocks mitochondrial fragmentation and mitochondrial release of cytochrome c and apoptosis. Mdivi-1, a derivative of quinazolinone acting as a selective inhibitor of Drp1 [141], blocks apoptotic cell death and drastically reduces the expression of cytochrome c [142, 143]. Overall, treatments targeting and manipulating cellular processes specifically associated with hepatic mitochondrial adaptation and bioenergetics may be implemented in the future to alleviate NAFLD and hence should be rigorously investigated for their therapeutic potential.

## Data Availability

Not applicable.

## Abbreviations

NAFLD: Non-alcoholic fatty liver disease
ATP: Adenosine triphosphate
OXPHOS: Oxidative phosphorylation
FAO: Fatty acid oxidation
BMI: Body mass index
TCA: Tricarboxylic acid cycle
ETC: Electron transport chain
ROS: Reactive oxygen species
IMM: Inner mitochondrial membrane
OMM: Outer mitochondrial membrane
Drp1: Dynamin-like/related protein 1
mtDNA: Mitochondrial DNA
FFA: Free fatty acid
TGs: Triglycerides
DNL: De novo lipogenesis
FATP: Fatty acid transport proteins
ER: Endoplasmic reticulum
HFD: High-fat diet
VLDL: Very low-density lipoproteins
NASH: Non-alcoholic steatohepatitis
